# Rare diagnosis of coexistent antiphospholipid syndrome and systemic lupus erythematosus in a male patient with successful management: a case report

**DOI:** 10.1097/MS9.0000000000000556

**Published:** 2023-04-06

**Authors:** Oadi N. Shrateh, Afnan W.M. Jobran, Azeeza Amoori

**Affiliations:** a Medical Research Club, Faculty of Medicine, Al-Quds University; b Faculty of Medicine, Al-Quds University, Jerusalem; c Al-Istishari Arab Hospital, Ramallah, Palestine

**Keywords:** antiphospholipid syndrome, autoimmune, systemic lupus erythematosus

## Abstract

Antiphospholipid syndrome (APS) and systemic lupus erythematosus (SLE) are two autoimmune disorders that can develop together or separately. Similarities in the pathogenesis have been discovered, including the production of autoantibodies that target subcellular antigens and shared elevated risk of cardiovascular morbidity, which may be caused by common pathologic pathways.

**Case presentation:**

A 28-year-old male, referred to our hospital for the assessment of chest pain. Past medical history was significant for extensive deep venous thrombosis despite the appropriate management with a therapeutic dose of direct-acting oral anticoagulant. Prolonged partial thromboplastin time was not corrected by mixing study along with positive lupus anticoagulant, anticardiolipin, and B-2 glycoprotein antibodies. In addition, antinuclear antibodies, anti-DNA antibodies, and direct Coombs were positive with decreased levels of C3. The patient was diagnosed with SLE with brain, heart, and kidney involvement in the setting of antiphospholipid antibody syndrome. He was treated successfully with full recovery.

**Discussion:**

SLE and APS both have sneaky ways of manifestation. Ineffective diagnosis and therapy could cause irreversible organ damage. Clinicians should have a high index of suspicion for APS, particularly in young patients who approach with spontaneous or unprovoked thromboses or unexplained recurrent early or late pregnancy loss. Anticoagulation, modifying cardiovascular risk factors, and identifying and treating any underlying inflammatory diseases are all part of the multidisciplinary care that is needed for management.

**Conclusion:**

Although male affection is rare, SLE and APS should be considered in male patients as these conditions tend to be more aggressive than in the female.

## Introduction

Highlights

- Antiphospholipid syndrome (APS) and systemic lupus erythematosus (SLE) are two autoimmune disorders that can develop together or separately.
- SLE and APS both have sneaky ways of manifestation. Therefore, organ damage that cannot be repaired may result from delayed diagnosis and therapy.
- Although they have a noticeable female predilection, Herein, we affirm the significance of suspecting SLE and APS in males due to the severe nature of clinical course and possible catastrophic sequelae in such patients.

The term “antiphospholipid syndrome” (APS) refers to the correlation between antiphospholipid antibodies (aPL) [lupus anticoagulant (LA), anticardiolipin antibodies (aCL), and/or anti-2-glycoprotein-I antibodies (a2GPI)] and thrombosis and/or pregnancy morbidity^1^. Chronic autoimmune disease, SLE can show as anything from moderate joint and skin involvement to potentially fatal cardiac, renal, hematologic, and/or central nervous system diseases^2^.

Primary APS, which occurs in otherwise healthy individuals without an underlying systemic autoimmune disease. In contrast, several other systemic autoimmune diseases, including SLE, can cause secondary APS. In addition, patients with SLE and/or APS might develop valvular heart disease, myocarditis, pulmonary hypertension, livedo reticularis/racemosa, thrombocytopenia, hemolytic anemia, renal thrombotic microangiopathy, and cognitive dysfunction^3^. Patients with SLE and/or aPL positivity may experience irreversible organ damage. Within 5 years of diagnosis, one-third of SLE patients get organ damage; similarly, one-third of primary APS patients with more than 10 years of disease experience organ damage^4^,^5^. Misdiagnosis of such a dangerous combination of SLE and APS could yield catastrophic and potentially fatal complications. This case report has been reported in line with the SCARE 2020 Criteria^6^.

## Case presentation

A 28-year-old male, referred to our hospital from the peripheral center for the assessment of chest pain. The patient mentioned that he started to complain an intermittent, central, and burning chest pain with radiation to the left shoulder for 5 days duration which was first evaluated as the outpatient clinic by computed tomography scan of the chest which ruled out pulmonary embolism. Past medical history was significant for two attacks of extensive deep venous thrombosis in the right lower limb; the first episode was before 8 months and the second one was 3 months ago despite of being on rivaroxaban. the patient’s sister had five recurrent pregnancy losses during the second trimester of each pregnancy. The patient reported no personal and/or family history of cancer, any acute, repeat, or discontinued medications, any allergies, any genetic or psychosocial issues, and had a free past surgical history. The pain did not relieve and progressed over days to be at rest with retrosternal burning nature.

Physical examination revealed a conscious, and alert patient with stable blood pressure and normal cardiac, pulmonary, and abdominal examinations. ECG showed sinus tachycardia with heart rate of 110 beats/min and ST-segment elevation in leads II and aVf along with elevated troponin levels. The patient underwent an urgent cardiac catheterization which was unremarkable. Echocardiography revealed reduced left ventricular systolic function with an ejection fraction of 30–35%, borderline left ventricular hypertrophy, lateral and inferior wall hypokinesia. Therefore, the diagnosis of myocarditis was established. Laboratory evaluation revealed a prolonged partial thromboplastin time of 105, mixing study was done and demonstrated no correction of prolonged partial thromboplastin time, positive LA, anticardiolipin, and B-2 glycoprotein antibodies. So, the patient was diagnosed with APS. On the second day of hospitalization, the complete blood count showed a sudden drop of hemoglobin levels from 16 to 11, platelet count from 170 to 69 with decreased serum albumin levels of 1.7. Urinanalysis showed proteinuria. Spot protein/creatinine showed nephrotic range proteinuria 4.5 g, hematuria, and granular casts. Reticulocyte count was increased of 3.4% and lactate dehydrogenase of 436. The patient was suspected to have another multisystemic condition. Further laboratory investigations including antinuclear antibodies, cytoplasmic-antineutrophil cytoplasmic antibodies, anti-DNA antibodies, and direct Coombs were positive along with decreased levels of C3 but normal C4. Blood film showed full of spherocyte compatible with autoimmune hemolytic anemia

**Figure 1:**
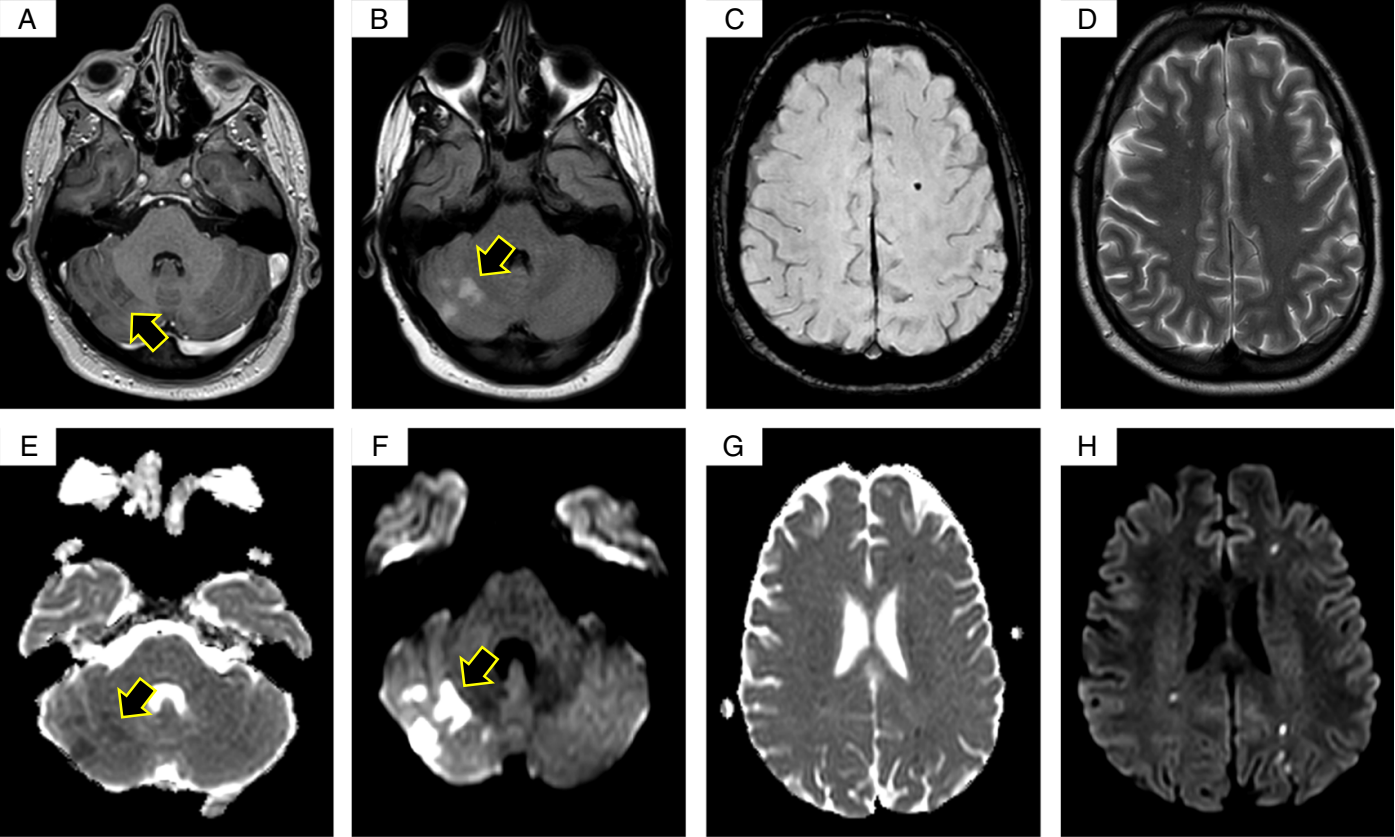
MRIs with different sequences as the following: T1 with contrast cerebellar enhancement (A), FLAIR (B), susceptibility-weighted image (C), T2 (D), DW2 (E and F), and diffusion-weighted images (G and H). Arrows indicate acute ischemic changes.

On the same day, the patient started to have a mild headache with blurred vision. Neuroimaging of the brain showed diffuse foci and areas of diffusion restriction in the centrum semiovale, subcortical white matter, and right cerebellar hemisphere reflecting hyperacute and acute ischemic insults (Fig. 1). The patient was diagnosed with SLEs involving the heart, brain, and kidney in the setting of APS. He was started immediately on a regimen consisting of pulse steroid therapy with a dose of 1 g once daily for 3 days followed by 1 mg/kg daily with tapering gradually 1 month later, and cyclophosphamide of 1000 mg once monthly for a duration of 6 months with planning to switch him to another immunosuppressant agent. He also was kept on hydroxychloroquine 400 mg twice daily and enoxaparin 1 mg/kg with bridging to warfarin. The patient was followed up for 4 months with significant improvement. The patient also had a good tolerance of chemotherapy and pharmacological agents without any reported complications or adverse events.

## Discussion

Autoantibodies that bind cell membrane phospholipids or phospholipid-binding proteins are present in the APS, and there may also be signs of arterial, venous, or small vessel thrombosis or obstetric problems^7^. Thrombotic consequences are numerous and may include splanchnic or cerebral vein thrombosis, myocardial infarction, peripheral thromboembolism, or uncommon locations for thrombosis^7^. Obstetric problems include repeated preterm birth due to placental insufficiency, late fetal loss, early-onset preeclampsia, and early pregnancy loss. A rare and potentially fatal variant of APS known as catastrophic APS manifests as several thrombotic problems almost simultaneously. Although APS may be primary, it is linked to a systemic autoimmune disorder, most frequently SLE, in more than one-third of patients^8^. Approximately 40% of SLE patients have these antibodies, and 20–50% of those who have the antibodies go on to develop the condition^7^,^8^.

aPL that is temporary is frequent during infections. It is crucial to have proof that an aPL was positive on two separate occasions that were at least a year apart^1^. Overall, 10% and 1% of healthy blood donors who underwent two aPL tests had baseline positive results for the aCL and LA tests, respectively. This data was based on prospective follow-up. Less than 1% of healthy blood donors tested positive for aCL or LA a year later^9^. Positive LA test compared with aPL enzyme-linked immunosorbent assay tests^10^, moderate to high aCL or a2GPI IgG/IgM titer (40 U or 99th percentile) compared with lower titers, and IgG or IgM isotype compared with IgA isotype^1^, and triple aPL (LA, aCL, and aβ_2_GPI) positivity (compared with single or dual aPL test positivity) correlate better with aPL-related clinical events^11^.

Independent of aPL, SLE patients are at much higher risk of early atherosclerosis and/or thrombosis due to the increased prevalence of classic cardiovascular disease and nontraditional lupus-related risk factors, such as inflammation, renal failure, or corticosteroids^12^. In general, vascular events are 10–30% common in SLE patients^13^, symptomatic coronary artery disease is 6–20%, stroke is 2–15%, and subclinical coronary artery disease is 30–40%^14^. According to a review of the Patient Discharge Database, women with SLE who are between the ages of 18 and 44 are hospitalized for myocardial infarction or stroke nearly twice as frequently as the general population^15^. Half of SLE patients^16^ experience anemia, which can be brought on by immunological or nonimmune causes. Autoimmune hemolytic anemia, the most prevalent form of immunological anemia in SLE, is present in 15% of SLE patients^17^.

While “aPL-nephropathy” affects between 4 and 16% of SLE patients who do not have an aPL, it affects 25–39% of those who do^18^. In the course of lupus nephritis, Tektonidou *et al.* 18 revealed that double aPL positivity (aCL and LA) is linked with aPL-nephropathy; more recently, Gerhardsson *et al.* 19 established that triple aPL positivity is associated with aPL-nephropathy in SLE patients. While thrombotic microangiopathy is acknowledged as a classification criterion for definite APS (small vessel thrombosis), the association between various aPL was inconsistent across studies, and it was unclear whether the frequency of chronic lesions was significantly higher in aPL-positive SLE patients compared with aPL-negative SLE patients. An updated meta-analysis of 1820 patients conducted by Domingues *et al.* 20 showed that the prevalence of renal lesions in aPL-positive versus aPL-negative SLE patients was 31.9%.

SLE has been linked to a wide range of neuropsychiatric symptoms. Only a small number of them, however, are more specific for SLE and useful in the diagnosis^21^,^22^. Seizures, psychosis, multiple mononeuritis, myelitis, peripheral or cranial neuropathy, and acute confusional condition are some of these^21^,^22^. Importantly, this demand ruling out other established reasons.

SLE primarily affects females of childbearing age, with a ratio of 9:1 female to male. However, the risk in women decreases after menopause, but it remains twice as high as in men. According to studies, lupus in men tends to be more aggressive and severe, despite being rare. Furthermore, men are more likely than women to have dermatologic manifestations, cytopenias, kidney disease, serositis, nervous system involvement, thrombosis, heart disease, hypertension, and vasculitis^23^. According to the aforementioned points, our case is considered outstanding, in which the patient was a male who achieved successful management and full recovery despite the severe clinical course he experienced, such as acute kidney injury.

## Conclusions

Both APS and SLE have sneaky ways of showing up. Organ damage that cannot be repaired may result from delayed diagnosis and therapy. Clinicians should have a high index of suspicion for APS, especially in young patients who report with unexplained recurrent early or late pregnancy loss or spontaneous or atypical thromboses. Although they have a noticeable female predilection, Herein, we affirm the significance of suspecting SLE and APS in males due to the severe nature of the clinical course and possible catastrophic sequelae in such patients. We also highlight the Importance of early recognition and management of these conditions that may alter the clinical outcomes largely.

## Ethical approval

Our institution has exempted this study from ethical review.

## Patient consent

Written informed consent was obtained from the patient for the publication of this case report and accompanying images. A copy of the written consent is available for review by the Editor-in-Chief of this journal on request.

## Sources of funding

This research did not receive any specific grant from funding agencies in the public, commercial, or not-for-profit sectors.

## Author contribution

O.N.S. and A.W.M.J.: writing the manuscript. A.A.: imaging description, and reviewing and editing the manuscript.

## Authorship

All authors attest that they meet the current ICMJE criteria for authorship.

## Conflicts of interest disclosure

The authors declare that they have no financial conflict of interest with regard to the content of this report.

## Research registration unique identifying number (UIN)

1. Name of the registry: None.
2. Unique Identifying number or registration ID: None.
3. Hyperlink to your specific registration (must be publicly accessible and will be checked): None.

## Guarantor

Oadi N. Shrateh.

## Provenance and peer review

Not commissioned, externally peer-reviewed.
